# Loneliness and adolescent mental health: a multilevel examination of socio-ecological factors across Czech schools

**DOI:** 10.1186/s13034-026-01026-3

**Published:** 2026-01-22

**Authors:** Zdenek Meier, Jakub Helvich, Lukas Novak, Jana Furstova, Vendula Machu, Lukas Vagner, Peter Tavel

**Affiliations:** 1 Olomouc University Social Health Institute, Palacky University in Olomouc, Univerzitni 244/22, Olomouc, 771 11 Czech Republic; 2 Department of Health Sciences, Community and Occupational Medicine, University Medical Center Groningen, University of Groningen, Groningen, The Netherlands

**Keywords:** Loneliness, Mental health, Adolescents, HBSC, School variance

## Abstract

**Background:**

Adolescent loneliness and mental health have become escalating public health concerns. However, despite previous findings, research on how the school environment influences the relationship between loneliness and mental health remains scarce.

**Objectives:**

Therefore, the objectives of this study are to identify key socio-ecological factors associated with adolescent mental health, examine the gender differences in socio-ecological factors and investigate whether the association between loneliness and mental health varies across individual schools.

**Methods:**

Data were drawn from the 2021/22 Czech dataset of the HBSC study, comprising 14,588 Czech adolescents aged 11–15 years old. Descriptive statistics and gender comparisons were conducted, followed by multilevel linear regression analyses accounting for the hierarchical structure of the data (students nested within schools). The nested models examined associations between mental health and key predictors using random intercepts and random slopes.

**Results:**

Boys reported better mental health, higher life satisfaction, stronger self-rated health and lower loneliness than girls. Boys also experienced better family support, communication and more frequent family meals. Girls reported more peer support, stronger preferences for online communication and greater academic pressure. Mental health was positively associated with family and health-related variables, and negatively with loneliness, bullying and academic stress. The relationship between loneliness and mental health was consistent across schools, with minimal variation attributable to school-level factors.

**Conclusions:**

While gender-based differences were observed, loneliness consistently showed a strong negative association with mental health for boys and girls. These findings emphasise the central role of individual and family-related factors in adolescent mental health. They also suggest that in more structurally and culturally homogeneous educational systems, school-level differences in mental health may be limited, with wider socioeconomic and cultural influences operating relatively uniformly across schools. This underscores the importance of system-wide and family-focused approaches as well as national school-based programmes.

## Introduction

 Adolescence is a critical developmental period marked by many physical, emotional and social changes, with approximately half of all mental health disorders emerging before the age of 14 [1, 2]. Furthermore, poor mental health in adolescence often persists into adulthood [3]. Recent evidence indicates a rising trend in adolescent mental health problems [4, 5], underscoring the urgent need to examine and address key risk factors along with broader socio-ecological factors that impact adolescents’ mental health. Among these factors, loneliness is particularly significant, as it has been consistently linked to a range of negative mental health outcomes, including increased risks of depression, anxiety and stress levels [6–10].

The present study aims to identify socio-ecological factors influencing adolescent mental health in the Czech context while focusing on gender differences, and to investigate school-level variations in the association between loneliness and mental health.

### Socio-ecological factors and mental health

Adolescent mental health is influenced by a complex interplay of individual, social, and environmental factors. Bronfenbrenner’s socio-ecological model [11] provides a valuable framework for understanding multiple levels of influence while recognising that individuals’ health is shaped by interactions of factors across different contexts, including family, schools, peers and wider community. Previous research has investigated several socio-ecological factors that shape adolescent mental health. Lower socioeconomic status has been associated with higher stress, limited access to mental health resources and increased vulnerability to loneliness and poor well-being [12–14]. Family communication and family support play crucial roles in adolescent mental health, with positive family communication, in particular, being linked to lower levels of depression and anxiety [15–18], whereas a lack of communication exacerbates feelings of loneliness [19, 20]. Similarly, strong family support serves as a protective factor against loneliness [20–22], providing emotional stability and reducing the likelihood of mental health issues [23–25]. Peer relationships are also critical in shaping adolescent mental health. Adolescents with low peer support often report increased feelings of loneliness and psychological distress [23, 24]. The school environment is another key factor, and poor school climate, characterised by high academic pressure and low emotional support, has been associated with poorer mental health [26–29]. In contrast, school connectedness, or a sense of belonging and engagement, helps promote well-being [30, 31], whereas adolescents who feel disconnected from school are more prone to experience loneliness [32–34]. Moreover, teacher support has been found to positively impact adolescent mental health, with strong student-teacher relationships associated with lower anxiety, depression and loneliness [35, 36]. Despite growing research on adolescent mental health, evidence from the Czech context is still limited, particularly with regard to examining factors across different levels of the socio-ecological framework.

Guided by Bronfenbrenner’s model and previous research, the present study examines the associations between mental health and socio-ecological factors on three levels of the microsystem: individual, social relationships and school environment. At the individual level, we examine loneliness, life satisfaction and self-rated health, i.e., factors that reflect personal perceptions and well-being. At the social relationship level, variables such as family communication and support, frequency of shared meals, peer support, experiences of bullying and engagement in online communication capture adolescents’ interpersonal relations. At the school level, factors such as teacher and classmate support, perceived school pressure and liking school capture the school climate. By examining factors across different levels, we align with Bronfenbrenner’s socio-ecological model, which emphasises the importance of considering how risk and protective factors interact rather than examining them in isolation. This approach allows us to better understand adolescent mental health determinants.

Additionally, adolescent girls and boys differ not only in the prevalence of mental health problems, which consistently show higher rates among girls [37], but also in how socio-ecological factors are associated with their mental health. Findings on gender differences in the relationship between socioeconomic disadvantage and adolescent mental health are mixed, with some studies indicating that a low-income background has a greater impact on girls [38], others suggesting stronger effects among boys [39] and some not finding any gender differences in adolescence [12]. At the family level, positive family relationships were associated with a greater reduction in depressive symptoms for females than for males during adolescence and early adulthood [15], whereas the association between a higher frequency of shared family dinners and prosocial behaviour was slightly stronger in males [17]. At the school level, the effect of stress related to schoolwork, peer exclusion and school context on psychosomatic symptoms and psychological distress was stronger in girls [40, 41]. Although the evidence of gender differences in the influence of socio-ecological factors on mental health is mixed, it highlights the need to investigate how these factors affect the mental health of adolescent girls and boys differently.

### Loneliness and mental health

Loneliness has become a growing public health concern [42, 43] due to its widespread and profound impact on both mental and physical well-being [44, 45], with over 80% of individuals under 18 reporting ever feeling lonely at school [42]. Loneliness has been associated with low self-esteem, negative self-perceptions and a greater propensity for self-harm, particularly among younger populations [46, 47]. Loneliness is defined as a subjectively felt lack of satisfying social relationships [42, 48]. According to Bronfenbrenner’s socio-ecological model, loneliness is the result of individual, interpersonal, community and societal influences. In other words, loneliness is shaped by a complex interplay of social, environmental and political factors and is not merely a personal matter [49–51].

Research indicates that loneliness is increasingly prevalent among young people, with those aged 16–24 being particularly vulnerable [52]. During adolescence, social networks undergo significant shifts, with peer relationships becoming more central while reliance on family support decreases [53–56]. The transition to peer relationships has been complicated by the COVID-19 pandemic, which intensified feelings of loneliness among adolescents, as lockdowns and social distancing measures disrupted their ability to interact with their peers [6, 46]. Across Europe, the prevalence of loneliness among adolescents rose sharply by 9% during the COVID-19 pandemic [57], but this pattern was not observed in all countries. For example, in Norway, loneliness showed a steady increase from 2014 to 2021, but no statistically significant additional rise was observed during the pandemic [58]. In other contexts, such as the Netherlands, loneliness levels returned to pre-pandemic values once restrictive policies ended [59].

While the Czech Republic fares comparatively well in overall loneliness rates in Europe, loneliness among adolescents and young adults increased fourfold between 2018 and 2022 [60]. Among school-aged children, the Czech Republic has experienced one of the most pronounced increases in loneliness over the past two decades [47]. While in countries with similar cultural characteristics, the prevalence of loneliness increased by an average of 49.62% between 2000 and 2018, the increase in the Czech Republic was substantially higher at 87.41%. This increase exceeded the loneliness rise observed in other countries in the region, such as Poland (20.83%), Austria (42.17%) and France (72.37%) [47]. Despite these trends, evidence on the consequences of the rise in loneliness among Czech adolescents, especially in the post-pandemic period, is still lacking.

The increase in loneliness is particularly concerning due to its well-researched link with mental health problems. Previous research showed a strong reciprocal association between loneliness and social anxiety [8] and depressive symptoms [61] in children and adolescents. Additionally, these associations differ by gender, with loneliness being more strongly associated with higher depression symptoms in girls and with higher social anxiety in boys [6]. While loneliness is experienced on an individual level, it is shaped by contextual factors, including the school climate. As schools play a key role in adolescents’ social lives, the variation in the school climate can contribute to differences in how loneliness and mental health interact.

### Between-school variation in loneliness and mental health

Given the significant rise in adolescent loneliness in the Czech Republic in recent decades [47], understanding how school-specific factors differ is crucial. Several school-level characteristics have been linked to heightened feelings of loneliness, including low teacher support and classroom management, poor peer cooperation, perceived teacher discrimination and higher levels of school-based victimisation [62]. School characteristics and experiences in school seem to be more important for explaining the incidence of loneliness than individual factors [63]. Therefore, variations in school-level factors, such as the overall school climate, the prevalence of academic pressure, the quality of teacher support and the availability of psychosocial resources, could plausibly moderate the strength of the relationship between loneliness and mental health. For instance, a supportive school climate might buffer the negative impact of loneliness, while a high-pressure environment could increase it.

In designing our research, we built upon the study by Goodfellow et al. [64], which was conducted under the international Health Behaviour in School-aged Children (HBSC) network. Goodfellow et al. [64] drew on 2018 data (i.e., pre-COVID-19 pandemic data) to explore inter-school differences in adolescent mental health in Scotland. However, no research to date has investigated school-level variation in the association between loneliness and mental health in the post-COVID-19 context, even though children and adolescents appeared particularly vulnerable to elevated levels of depression and anxiety during and following periods of enforced isolation [6]. Therefore, this study aims to address the gaps in the literature by examining whether the strength of the association between loneliness and mental health varies across schools using data collected in 2022. Identifying school-level differences in the association between loneliness and mental health may provide a deeper understanding of the role contextual factors play in adolescent well-being.

The aims of this study are therefore threefold:

1. Identify key socio-ecological factors associated with adolescent mental health.
2. Examine gender differences in socio-ecological factors.
3. Investigate whether the association between loneliness and mental health varies across individual schools.

## Methods

### Participants and procedure

This study is based on data from the 2021/22 wave of the Health Behaviour in School-aged Children (HBSC) study conducted in the Czech Republic. The HBSC is an international cross-sectional survey carried out every 4 years in collaboration with the World Health Organization to assess health, lifestyle and related social factors among 11-, 13- and 15-year-old adolescents (http://www.hbsc.org*).* Data collection took place between May and June 2022. Schools were randomly chosen using stratified sampling based on region, size and educational level (primary or secondary). Of the 272 schools contacted, 246 agreed to participate in the study, resulting in a school response rate of 86.1%. A class was randomly selected from each of the 5th, 7th and 9th grades (generally corresponding to ages 11, 13 and 15), making up a total sample of 14,879 pupils and an overall response rate of 83.1%. For the present study, an additional 111 respondents were removed due to unreliable responses, such as mutually exclusive or nonsensical answers to open-ended questions, and 12 respondents were discarded due to excessive missing data. Additionally, a total of 168 respondents were removed from the sample due to age outside the permitted range (ages 11–15). The Czech dataset includes responses from 14,588 participants (mean age 13.6 ± 1.7 years; 49.3% girls).

Data collection followed the international HBSC study protocol [65], ensuring methodological consistency. Participation was entirely voluntary and anonymous, with no financial or other incentives offered. Informed consent procedures followed national ethical guidelines: consent was either obtained directly from parents or guardians, or indirectly through school principals in cases where parents had previously granted general consent for participation in school-related surveys and activities. The study was approved by the ethics committee of the Faculty of Physical Culture at Palacký University Olomouc (Approval No. 14/2019) and conducted in accordance with the Declaration of Helsinki.

### Measures

*Mental health* was assessed using the psychological complaints subscale of the Multiple Health Complaints (MHC) scale [65], an eight-item instrument comprising four psychological and four somatic symptom items. The psychological complaints subscale includes four items: feeling low, irritability or bad mood, nervousness, and sleep difficulties. These were rated on a five-point scale, ranging from 1 (about every day) to 5 (rarely or never). A summary score was calculated and used in the analyses, with higher scores indicating better mental health. The psychological complaints subscale has been associated with indicators of emotional well-being and may serve as an early marker of more serious mental health issues that can emerge in young adulthood [66]. In the present study, the internal consistency was satisfactory (α = 0.797, 95% CI 0.791–0.802).

*Demographic variables* included age, gender and socioeconomic status (SES). SES was assessed using the Family Affluence Scale III (FAS-III), a six-item measure of household material wealth and lifestyle indicators (including having a personal bedroom, the number of cars in the household, holidays abroad, the number of computers, the number of bedrooms and the presence of a dishwasher [67]). Each item contributes to a total score ranging from 0 to 13, with higher scores indicating greater affluence. The FAS-III has been shown to be a valid and age-appropriate indicator of SES, particularly suitable for use with younger populations [67].

### Individual factors

*Self-rated health* was assessed using a single item rated on a four-point scale, with higher values corresponding to better perceived health. This global measure captures an adolescent’s holistic perception of their well-being, which is distinct from the specific symptomatology assessed by the psychological complaints scale.

*Life satisfaction* was evaluated using the Cantril Self-Anchoring Striving Scale [68]. The participants were asked to assess their life on a scale from 0 (worst possible life) to 10 (best possible life). A study by Levin and Currie [69] demonstrated that the measure exhibits good reliability in adolescents aged 11 to 15 years.

*Loneliness* was assessed using a single item assessing perceived general loneliness over the past 12 months. This approach has been previously utilised in large-scale international surveys to estimate the prevalence of loneliness among youth populations [70]. Participants responded using a five-point scale ranging from 1 (never) to 5 (always), with higher scores indicating greater perceived loneliness. Prior research has shown that single-item measures of loneliness exhibit reliability and validity comparable to multi-item scales [71], with similar associations observed between loneliness and health outcomes [72].

### Social-relationship variables

*Family communication* was assessed using two items evaluating how easily participants could talk to their mother and father about important matters. Each item was scored on a four-point scale from 1 (very difficult) to 4 (very easy). A summary score was then created in which higher values denoted greater ease of family communication. *Shared family meals* were assessed with one item rated on a five-point scale ranging from 1 (never) to 5 (every day); it was reverse coded for analysis.

*Perceived family and peer support* were assessed using the respective four-item subscales of the Multidimensional Scale of Perceived Social Support [73], rated on a seven-point Likert scale (1 = very strongly disagree, 7 = very strongly agree). An example item is “*My family really tries to help me.*” Summary scores range from 4 to 28, with higher scores indicating higher levels of perceived social support. In the present study, the internal consistency was high (α = 0.933, 95% CI 0.931–0.934 for family support; α = 0.927, 95% CI 0.925–0.928 for peer support).

*Bullying victimisation* was assessed using a single item adapted from the Olweus Bully/Victim Questionnaire [74]. Response options ranged from 1 (I have not been bullied) to 5 (several times a week). The Olweus Bully/Victim Questionnaire is one of the most validated instruments for measuring school bullying [75].

*Social media engagement* was measured through *frequency of online contact* and *preference for online communication*. Intensity of electronic media communication (EMC) was captured using four items that assessed how often participants interacted online with: close friends, members of a broader friend group, individuals met online but not known in person and other non-friend individuals. Each item was rated on a five-point scale ranging from 1 (never or almost never) to 5 (almost all the time throughout the day). A summary score was created to assess the overall intensity of EMC, where higher scores indicate higher EMC intensity. Preference for online social interaction (POSI) was assessed using a single item: “I talk more easily about my inner feelings than in a face-to-face encounter”, rated on a five-point Likert scale from 1 (strongly disagree) to 5 (strongly agree). Higher scores indicate a stronger preference for online communication.

### School factors

*Perceived social support from teachers and classmates* was assessed using the corresponding three-item subscales from the Teacher and Classmate Support Scale [76]. An example item for teacher support is “*I feel that my teachers accept me as I am*” and for classmate support “*The students in my classes enjoy being together*.” Items were rated on a five-point scale ranging from 1 (strongly agree) to 5 (strongly disagree). After reversing the coding, the scores were summarised to create an overall score of teacher/classmate support, with higher values corresponding to greater perceived social support. In the present study, the internal consistency was satisfactory (α = 0.812, 95% CI 0.807–0.818 for teacher support; α = 0.778, 95% CI 0.771–0.784 for classmate support).

In addition, two items were used to measure school satisfaction. *Liking school* was rated on a four-point scale from 1 (not at all) to 4 (like it a lot). *Perceived school pressure* was rated on a four-point scale from 1 (no pressure) to 4 (a lot of pressure).

### Data analysis

Descriptive statistics were computed to summarise the characteristics of the study sample. Independent variables considered for subsequent regression modelling were compared between gender groups using Welch’s t-test, which accounts for unequal variances. In addition to p-values corresponding Cohen’s d effect size estimates were reported. Spearman’s rank-order correlation coefficients were calculated to assess bivariate relationships among the study variables.

To account for the natural hierarchical structure of the data and potential between-school variability, multilevel linear regression analyses were conducted to examine the links between mental health, individual/demographic, social-relationship and school-related factors. A series of nested models were estimated, with mental health as the dependent variable. Individual/demographic, social relationships and school-related factors were gradually added as independent variables. All models were controlled for demographic variables. Individuals (Level 1) were nested within schools (Level 2), and random intercepts were specified for all partial models.

To investigate whether the relationship between loneliness and mental health differs across schools, we included a random slope for loneliness in the final, full model, allowing its association with mental health to vary across schools. To facilitate interpretation and comparison of regression coefficients, all continuous independent variables were standardised prior to analysis. In the tables, b coefficients represent unstandardised coefficients, and β represent standardised coefficients. Multicollinearity was assessed in each model using variance inflation factors (VIFs) to ensure that the independent variables did not exhibit problematic collinearity.

All statistical analyses were performed in the R software, version 4.4.1 (R Core Team, 2024), within the RStudio environment, version 2024.04.2. Multilevel models were estimated using the *lme4* package [77].

## Results

### Identification of socio-ecological correlates of adolescent mental health

As shown in Table 1, mental health exhibited significant correlations (*p* < 0.001) with all the examined variables, except for the socioeconomic status (*r* = 0.01). The strongest negative correlations were observed with loneliness (*r* = − 0.60) and academic pressure (*r* = − 0.45). Conversely, the strongest positive correlations were found with life satisfaction (*r* = 0.49), family support (*r* = 0.40) and family communication (*r* = 0.39).

**Table 1:** Spearman (non-parametric) bivariate correlations between mental health, demographic, individual, social-relationship and school factors in the HBSC 2022 Czech Republic sample

Variables	1	2	3	4	5	6	7	8	9	10	11	12	13	14	15	16	17
1. Mental health																	
2. Loneliness	− 0.60*																
3. Sex	− 0.29*	0.30*															
4. Age	− 0.11*	0.13*	− 0.06*														
5. SES	0.01	− 0.04*	− 0.03	0.04*													
6. Self-rated health	0.37*	− 0.32*	− 0.14*	− 0.04*	0.11*												
7. Life satisfaction	0.49*	− 0.47*	− 0.17*	− 0.16*	0.12*	0.42*											
8. Family communication	0.39*	− 0.39*	− 0.20*	− 0.12*	0.07*	0.26*	0.37*										
9. Family support	0.40*	− 0.39*	− 0.11*	− 0.17*	0.06*	0.28*	0.45*	0.48*									
10. Family meal	0.28*	− 0.27*	− 0.11*	− 0.21*	0.09*	0.21*	0.29*	0.27*	0.32*								
11. Peer support	0.16*	− 0.18**	0.10*	− 0.01*	0.04*	0.16*	0.22*	0.18*	0.35*	0.09*							
12. Been bullied	− 0.22*	0.20*	0.02	− 0.02*	− 0.02	− 0.16*	− 0.16*	− 0.11*	− 0.16*	− 0.07*	− 0.15*						
13. Online contact	− 0.11*	0.06*	0.02	0.16*	0.08*	− 0.03	− 0.03	0.04*	0.00	− 0.03	0.17*	0.04*					
14. Preference for online communication	− 0.28**	0.26*	0.10*	0.23*	0.01	− 0.14*	− 0.23*	− 0.23*	− 0.24*	− 0.15*	− 0.03*	0.09*	0.24*				
15. Teacher support	0.32*	− 0.28*	− 0.06*	− 0.21*	− 0.03*	0.22*	0.32*	0.26*	0.37*	0.23*	0.23**	− 0.12*	− 0.06*	− 0.17*			
16. Like school	0.30*	− 0.24*	0.03*	− 0.15*	− 0.01	0.19*	0.28*	0.19*	0.25*	0.19*	0.20*	− 0.16*	− 0.06*	− 0.12*	0.43*		
17. School pressure	− 0.45*	0.35*	0.18*	0.12*	0.00	− 0.22*	− 0.30*	− 0.25*	− 0.25*	− 0.16*	− 0.11*	0.15*	0.06*	0.19*	− 0.29*	− 0.30*	
18. Classmate support	0.28*	− 0.27*	− 0.10*	− 0.01	0.03*	0.22*	0.27*	0.21*	0.26*	0.16*	0.30*	− 0.28*	0.04*	− 0.07*	0.38*	0.37*	− 0.21*

*p < 0.001; Variables in the columns are numbered: the same numbers in row 1 correspond to the variables listed in column 1 (i.e., number 1 in the row = Mental health in column 1, etc)

### Gender differences in socio–ecological factors

At the individual level, boys reported higher mental health scores and lower levels of loneliness compared to girls (both *p* < 0.001, with a medium effect size of d = 0.6). Additionally, boys reported better overall health and greater life satisfaction (*p* < 0.001), though the effect sizes were small (d = 0.27 and d = 0.35, respectively).

In terms of social relationships, boys reported better family communication, greater family support and more frequent family meals (*p* < 0.001, with small effect sizes). In contrast, girls reported higher levels of peer support and a stronger preference for online communication (*p* < 0.001), though the effect sizes were small or trivial. No significant differences or only trivial effect sizes were observed between boys and girls in the frequency of being bullied or online contact.

In the school context, boys reported greater teacher and classmate support, whereas girls exhibited higher scores for liking school and were more likely to report experiencing academic pressure (all *p* < 0.001, with small or trivial effect sizes). For a detailed comparison between boys and girls, refer to Table 2.

**Table 2:** Descriptive characteristics of the study sample, including mental health and socio-ecological factors, overall and stratified by sex

Variables	Boys	Girls	*p* value	Effect size
Total: N (%)	7395 (50.7)	7193 (49.3)		
Dependent variable: mean (SD)				
Mental health [0–16]	10.5 (4.04)	8.01 (4.44)	< 0.001	0.59
Independent variables: Mean (SD)				
Demographics				
Age [years]	13.7 (1.66)	13.6 (1.67)	< 0.001	0.10
SES [0–13]	8.32 (2.28)	8.19 (2.29)	< 0.001	0.06
Individual factors				
Self-rated health [1–4]	3.19 (0.701)	3.00 (0.713)	< 0.001	0.27
Life satisfaction [0–10]	7.91 (1.76)	7.24 (2.02)	< 0.001	0.35
Loneliness	2.19 (1.06)	2.84 (1.05)	< 0.001	0.61
Social relationships				
Family communication [2–8]	6.13 (1.66)	5.44 (1.74)	< 0.001	0.40
Family meal [1–5]	3.67 (1.15)	3.43 (1.16)	< 0.001	0.21
Family support [4–28]	22.8 (6.56)	21.3 (7.10)	< 0.001	0.22
Peer support [4–28]	20.0 (6.74)	21.2 (6.73)	< 0.001	0.18
Been bullied [1–5]	1.37 (0.905)	1.36 (0.860)	< 0.001	0.00
Online contact [2–20]	9.25 (3.93)	9.27 (3.66)	0.86	0.00
Preference for online communication [1–5]	2.15 (1.29)	2.40 (1.34)	< 0.001	0.20
School factors				
Teacher support [0–12]	7.30 (2.83)	7.02 (2.72)	< 0.001	0.10
Classmate support [0–12]	7.38 (2.71)	6.89 (2.63)	< 0.001	0.18
Like school [1–4]	2.56 (0.822)	2.62 (0.783)	< 0.001	0.07
School pressure [1–4]	2.35 (0.915)	2.68 (0.914)	< 0.001	0.36

Health behaviour in School-aged children study, Czech Republic, 2022

Differences between boys and girls are tested using Welch’s t-test, effect size is measured with Cohen’s d

### Multilevel analysis: associations between loneliness and mental health, accounting for socio-ecological factors and school-level variation

The associations of demographic, individual, social and school factors with mental health are presented in Table 3. Several nested regression models were performed. Model 1 assessed the unadjusted association between loneliness and mental health (β= − 0.61, *p* < 0.001), indicating that higher loneliness was associated with a lower mental health score. In Model 2, which included individual and demographic predictors, loneliness demonstrated the strongest association with mental health (β= − 0.42, *p* < 0.001). Additionally, girls (compared with boys) reported poorer mental health (β= − 0.21, *p* < 0.001), while better self-rated health (β = 0.14, *p* < 0.001) and higher life satisfaction (β = 0.23, *p* < 0.001) were linked with better mental health outcome. Age and socioeconomic status had very low effect sizes (β= − 0.03 and β= − 0.05, respectively) and were therefore considered negligible.

**Table 3:** Summary of linear mixed-effect regression analysis with mental health as the dependent variable in the HBSC 2022 Czech Republic sample: four nested models (Model 1–4) progressively including individual/demographic, social-relationship and school factors

Independent variables	Model 1		Model 2		Model 3		Model 4		Model 4 with random slopes	
	b	β	b	β	b	β	b	β	b	β
Fixed effects										
Loneliness	− 2.44*	− 0.61*	− 1.67*	− 0.42*	− 1.44*	− 0.36*	− 1.28*	− 0.32*	− 1.28*	− 0.32*
Demographics/individual factors										
Gender [girls vs. boys]			− 0.95*	− 0.21*	− 0.99*	− 0.22*	− 0.82*	− 0.19*	− 0.82*	− 0.19*
Age			− 0.08*	− 0.03*	0.04	0.01	0.08*	0.03*	0.08*	0.03*
SES			− 0.10*	− 0.05*	− 0.09*	− 0.05*	− 0.08*	− 0.04*	− 0.08*	− 0.04*
Self-rated health			0.86*	0.14*	0.66*	0.11*	0.57*	0.09*	0.57*	0.09*
Life satisfaction			0.53*	0.23*	0.41*	0.18*	0.34*	0.15*	0.34*	0.15*
Social relationships										
Family communication					0.25*	0.10*	0.20*	0.08*	0.20*	0.08*
Family support					0.04*	0.05*	0.03*	0.05*	0.03*	0.05*
Family meal					0.19*	0.05*	0.16*	0.04*	0.16*	0.04*
Peer support					0.01	0.02	0.00	0	0.00	0
Been bullied					− 0.31*	− 0.06*	− 0.24*	− 0.05*	− 0.24*	− 0.05*
Online contact					− 0.09*	− 0.07*	− 0.07*	− 0.06*	− 0.07*	− 0.06*
Preference for online communication					− 0.18*	− 0.05*	− 0.14*	− 0.04*	− 0.14*	− 0.04*
School factors										
Teacher support							0.02	0.01	0.02	0.01
Like school							0.26*	0.05*	0.26*	0.05*
School pressure							− 0.93*	− 0.20*	− 0.93*	− 0.20*
Classmate support							0.02	0.01	0.02	0.01
Random effects										
Residual σ^2^	12.26		10.47		9.55		8.78		8.78	
School intercept variance	0.05		0.05		0.07		0.05		0.01	
School slope variance (loneliness)									0.002	
ICC	0.004		0.005		0.007		0.006		0.006	
Model performance										
Observations	13,076		12,538		9238		9078		9078	
AIC	69,941.4		65,106.1		47,149.2		45,571.1		45,574.5	
BIC	69971.3		65,173.0		47,263.3		45,713.4		45,731.0	
Marginal R^2^/Conditional R^2^	0.371/0.374		0.463/0.465		0.504/0.507		0.543/0.546		0.543/0.546	

The final model includes a random slope for loneliness to account for between-school variation

* *p* < 0.001; b coefficients represent unstandardised estimates; Continuous variables were standardised to compute β coefficients; A check for multicollinearity revealed all VIF values < 1.75, showing no multicollinearity issue; the clustering variable comprised 246 schools

Subsequent models incorporated social relationship variables (Model 3) and school-related factors (Model 4), with the results for individual and demographic predictors remaining largely consistent. In Model 3, variables such as family communication and frequency of family meals were positively associated with mental health, whereas experiences of bullying and a preference for online communication were negatively associated. However, their effect sizes were generally small (< 0.1), except for family communication (β = 0.10, *p* < 0.001). In the final model (Model 4), the strongest negative associations of mental health were with loneliness (β= − 0.32, *p* < 0.001), school pressure (β= − 0.2, *p* < 0.001) and female gender (β= − 0.19, *p* < 0.001), while the strongest positive association was with life satisfaction (β = 0.15, *p* < 0.001).

To account for the clustering of adolescents within schools, random effects were examined. The random intercept variance was consistently small across all models (ranging from 0.01 to 0.07). Furthermore, in Model 4, the random slope variance for loneliness was low (0.002), suggesting that the strength of the association between loneliness and mental health did not vary substantially across schools. Additionally, the ICC ranging between 0.004 and 0.007 indicated that only about 0.4–0.7% of the total variance in mental health was attributable to differences between schools. For further details, see Table 3.

## Discussion

The objectives of the study were: first, to identify key socio-ecological factors that are associated with adolescent mental health. Second, we aimed to investigate gender differences in socio-ecological factors and their associations with adolescent mental health. Third, we examined whether the association between loneliness and mental health varies across schools. Across the board, mental health was significantly correlated with nearly all the examined variables, revealing the strongest positive correlation with life satisfaction, self-rated health, family support, frequency of family meals and family communication, while the strongest negative associations were observed with loneliness, academic pressure, experiences of bullying and preference for online communication. Additionally, the findings revealed several differences between genders. On the personal level, boys reported higher mental health scores, better overall health, greater life satisfaction and lower levels of loneliness compared to girls. On the social level, boys reported better family communication, greater family support and more frequent family meals. On the other hand, girls reported higher levels of peer support and a stronger preference for online communication. On the school level, boys reported greater teacher and classmate support. In contrast, girls were more likely to report experiencing academic pressure. Notably, the relationship between loneliness and mental health remained consistent across schools, with only a very small percentage of mental health variability attributable to school-level differences.

### Socio-ecological factors and adolescent mental health

Meta-analytic evidence indicates that adolescents with high life satisfaction experience substantially fewer mental health issues [61]. Longitudinal studies further suggest that life satisfaction and mental health mutually reinforce each other over time [78]. This bidirectional linkage implies that life satisfaction not only coexists with better mental health but also promotes it, while positive mental health helps youths perceive their lives more favourably. Prior research also shows that self-rated health strongly relates to both physical and psychological well-being [79]. Indeed, previous studies suggest that mental health is a stronger predictor of adolescents’ self-rated health than objective somatic indicators [79, 80]. Conversely, lonely adolescents are far more prone to experience depression, anxiety and low life satisfaction [61, 81]. What’s more, longitudinal evidence confirms that the relationship is reciprocal: loneliness can precipitate subsequent depression, and conversely, depressed individuals often become more socially withdrawn and lonely over time [81]. Adolescents who prefer online social interaction via social media rather than in person often do so because of underlying loneliness, social anxiety or poor mental health [82, 83]. Conversely, those who spend excessive time on social media sites tend to experience more psychological distress, including depression and self-harm thoughts, forming a reciprocal loop [84]. Bullying victimisation also showed a strong negative association with mental health, as being bullied is a known risk factor for depression, anxiety, low self-worth and suicidal ideation [85–87]. Victims additionally report more psychosomatic complaints and loneliness [85, 88], while adolescents with pre-existing emotional vulnerabilities are more likely to be targeted, increasing their risk of further victimisation [88]. In contrast, adolescents who can confide in their parents, feel understood and supported and engage in regular family interactions exhibit fewer depressive and anxiety symptoms [18, 89]. In our sample, frequent family communication and shared meals were associated with better mental health, consistent with evidence linking routine family meals to fewer depressive symptoms and greater life satisfaction [90]. The relationship between family factors and adolescent mental health is likely bidirectional: supportive family environments foster better mental health, while adolescents’ psychological states can influence family dynamics [91]. These patterns are consistent with Bronfenbrenner’s socio-ecological model, which conceptualises adolescent mental health as emerging from ongoing reciprocal interactions between individual characteristics and proximal social contexts, particularly close family relationships [11]. Altogether, each correlation observed in our study represents not a static association, but part of a larger reciprocal feedback loop consistently documented in prior research.

### Gender differences in socio-ecological factors

Our finding of a significant gender gap, where boys reported better mental health, higher life satisfaction and lower loneliness, aligns with extensive international research. This body of work indicates that adolescent girls typically report higher psychosocial distress, lower well-being and more loneliness than their male peers [92–94], with evidence suggesting that the gender gap often widens during adolescence [92, 95]. Factors such as hormonal changes, body image issues, academic pressures and differences in coping and help-seeking behaviours are often suggested to contribute to these disparities [95]. Conversely, boys’ lower reported loneliness may positively correlate with their higher life satisfaction and better self-rated health, highlighting the interconnectedness of these factors [96–98]. These observations are consistent with the findings by Goodfellow et al. [64], who observed that higher self-rated physical health and life satisfaction are related to better mental health, while loneliness is strongly, and negatively, associated with mental health. One explanation could be that although girls generally maintain stronger social connections [99, 100], they tend to be more sensitive to social stressors and relational complexities [100], contributing to their increased loneliness. Altogether, the evidence has consistently shown a significant multifaceted gender disparity in adolescent mental health, predominantly disfavouring girls. This disparity, however, might be partly a function of how mental health is operationalised. Girls appear more inclined than boys to perceive, monitor and report internal states and relational stressors, and they score higher on self-report instruments capturing internalising or socio-emotional dissatisfaction, whereas boys more often under-report such problems or externalise distress, take part in disturbances or substance-related risks, and experience injuries or mortality due to accidents and violence [37, 95, 101, 102]. This well-described “gender paradox” does not necessarily mean that girls are the group with the most severe or most lethal mental-health issues; rather, each gender tends to express psychological burden through different symptom channels, which are captured differentially by commonly used survey instruments.

Some recent studies have similarly observed that girls often report higher peer support but more difficulty communicating with parents compared to boys [103, 104]. Additionally, a number of national surveys found boys were more likely to have frequent family dinners than girls [105, 106]. These international findings tightly coincide with the present data, suggesting that Czech adolescent boys generally maintain more regular family engagement, whereas girls may distance themselves from family routines. This gender disparity in family connectedness is concerning, highlighted by our previous observations, where family support, frequency of family meals and family communication were positively correlated with mental health, while a preference for online communication was correlated negatively. These correlational results are akin to Goodfellow et al. [64] who demonstrated that both an increased frequency of online contact and a strong preference for online communication were associated with worse mental health. Several large-scale studies reported a strikingly similar pattern that girls, compared to boys, are more likely to engage in frequent online chatting as an extension of their peer relationships [107, 108]. This resonates with the growing body of work linking high reliance on digital social interaction to increased psychosocial problems and feelings of loneliness [109–111]. Taken together, these findings underscore another dimension where girls are often disadvantaged.

Recent research has consistently shown that boys tend to report higher levels of teacher and classmate support, while girls experience greater academic pressure [26, 112, 113]. For example, a large European survey showed nearly half of 15-year-old boys, but only one-third of girls, felt strongly supported by teachers [104]. The same survey indicated almost two-thirds of girls, compared to less than half of boys, reported intense academic pressure [104]. These gender differences may stem from girls often internalising higher expectations and striving for perfection, which may correlate with increased academic pressure [26], while boys tend to be more laid back [114]. Studies confirm that girls experience more school-related stress [115, 116] and face more pressure to achieve high standards compared to boys [114, 117]. This aligns with our findings demonstrating a strong negative correlation between academic pressure and mental health. Additionally, research indicates boys generally report a more supportive classroom peer climate [113], while girls depend more on close friends for emotional support [99, 100] yet lack broader class, family and teacher support [103, 104]. Our results also converge with the findings of Goodfellow et al. [64], suggesting that adolescents who perceived strong teacher support had better mental health outcomes on average, while those experiencing high school-related pressure had worse mental health. Overall, evidence consistently shows adolescent girls perceive less school support but higher academic pressure, whereas boys report the opposite balance of lower pressure and more school support.

### Between-school variation in mental health and differing association with loneliness

Notably, our results indicate that school-level factors play a minimal role in the overall variance of adolescent mental health in our Czech sample. The Intraclass Correlation Coefficient (ICC) was very low across all models (ICC < 0.007), suggesting that less than 1% of the total variability in mental health scores can be attributed to differences between schools. Furthermore, the association between loneliness and mental health remained consistent across schools, as evidenced by the negligible random slope variance (0.002). Taken together, these findings suggest that in our Czech sample school-based contexts may not play a pivotal role in shaping the mental health of children and adolescents. Our finding thus contrasts with the research study from Scotland [64], where notable between-school differences in mental health were observed. Economic inequality offers one plausible explanation for this discrepancy. The Gini coefficient (GC)—a standard index of income inequality where 0 represents a perfect equality and 1 represents perfect inequality—shows that the Czech Republic has a more even income distribution (GC: 0.244) than Scotland (GC: 0.31) [118]. Research also demonstrates that higher inequality is tied to poorer child mental health [119, 120]. Therefore, in Scotland, pupils from wealthier areas may fare better psychologically than their peers in low‑income areas, producing school‑to‑school disparities. In contrast, in the Czech context, broader socioeconomic influences may act more uniformly, dampening school‑level variation. Beyond socioeconomic factors, the structural homogeneity of the Czech educational system may also play a role. Even though the Czech education system is highly decentralised [121], there are relatively standardised school policies according to which children are prepared for unified high school entrance exams [121]. This could lead to less variability in school climate, academic pressure and the availability of support services across schools, thus dampening potential school-level effects. Finally, from a cultural perspective, our findings may also reflect deeper cross-national differences in educational and social values. According to Hofstede’s cultural dimensions, the Czech Republic scores significantly higher on uncertainty avoidance (UAI = 74) and lower on individualism (IDV = 58) than the United Kingdom (UAI = 35; IDV = 89) [122–124]. These cultural characteristics suggest that Czech adolescents are socialised in a context that values stability, predictability and conformity, which may lead to schools functioning in a more homogeneous manner in terms of climate and expectations [125]. In contrast, Scottish schools, embedded in a more individualistic culture, often emphasise autonomy, local decision-making and personal responsibility, leading to greater variability in the psychosocial environment and student well-being across schools [126, 127]. This cultural uniformity, combined with the structural standardisation of Czech education, likely contributes to the minimal differences at the school level that we observed in our study.

### Limitations

Our study has several limitations that should be declared. First, the cross-sectional design of the present study limits the ability to infer causality between loneliness, mental health and socio-ecological factors. Second, the reliance on self-reported measures introduces the possibility of response bias. Participants may underreport or overreport their feelings of loneliness and mental health complaints due to factors such as social desirability bias or recall bias, potentially affecting the accuracy of the findings. Third, the study is limited to a single country, the Czech Republic, which may restrict the generalisability of the results to other cultural contexts where social norms, support systems and perceptions of loneliness differ. Finally, our study also utilised some single-item measures to assess loneliness, mental health and socio-ecological factors. While such measures are more practical for large-scale surveys and have demonstrated acceptable validity and reliability, they may lack the sensitivity to capture nuanced aspects of these constructs, such as the distinction between emotional and social loneliness or different dimensions of mental well-being.

### Implications

#### Implications for research

Several implications for future research follow from the current study. First, future research should extend our findings by incorporating longitudinal and experimental designs to disentangle causal directions between loneliness and mental health in adolescence. Second, conducting multi-country studies could clarify what factors modulate the variability in mental health across schools in different countries. Third, researchers may also benefit from employing more comprehensive measures of loneliness (e.g., multidimensional scales) and mental health (e.g., well established instruments capturing emotional, psychological and social well-being) to capture nuanced differences that single-item measures might overlook. Fourth, including vulnerable subgroups, such as immigrant and LGBTQ + adolescents, could further enhance understanding of how intersectional factors shape loneliness and mental health outcomes. Finally, mixed-method studies incorporating qualitative perspectives would provide deeper insight into the lived experiences of adolescents, offering clues about the mechanisms driving loneliness and mental health across different socio-ecological contexts.

#### Implications for practice

Given that school-level differences in loneliness-related mental health outcomes appear to be minimal (at least in the Czech Republic context), the findings suggest a high degree of homogeneity across Czech schools. Consequently, schools remain ideal settings for implementing universal interventions, as they provide access to the vast majority of adolescents and remain a critical context for socialisation. Interventions strengthening open communication and supportive relationships within families should complement these school-based efforts. Schools can still play a vital part by fostering awareness and training for teachers, enabling them to recognise signs of loneliness and mental distress early and providing appropriate referrals or support. Furthermore, as our findings indicated that individual perceptions of school pressure and support significantly impact mental health, efforts to improve the general school climate remain essential, even if these factors do not vary substantially between different schools. Community-based initiatives, such as youth clubs or mentorship programmes, can also offer social connections that mitigate loneliness outside the school setting. Additionally, campaigns raising awareness about responsible digital engagement could help adolescents balance online interactions with in-person relationships. Collectively, these efforts underscore the importance of a multi-pronged approach, where families, schools and community resources align to support adolescent mental health and counteract the possible detrimental impact of loneliness.

## Conclusion

The objectives of the study were to identify key socio-ecological factors that are associated with adolescent mental health, to investigate gender differences in socio-ecological factors and their associations with adolescent mental health and to examine whether the association between loneliness and mental health varies across schools. The findings have highlighted significant gender differences across personal, social and school-related levels. Boys generally reported better mental health outcomes, lower levels of loneliness and stronger support systems within the family and school environments. In contrast, girls experienced higher peer support and a greater inclination toward online communication yet also reported greater academic pressure. Across the entire sample, mental health was associated with a wide range of socio-ecological variables. Positive mental health outcomes were most strongly associated with higher life satisfaction, better self-rated health, strong family support, regular family meals and effective family communication. Conversely, loneliness, academic pressure, experiences of bullying and a preference for online communication were among the strongest negative correlates of mental health. These findings underscore the importance of addressing both individual and contextual factors—particularly family dynamics and social connectedness—in efforts to support adolescent mental health. While family-level engagement is crucial, schools remain indispensable platforms for delivering these interventions, particularly universal programmes that foster a supportive climate and reduce academic pressure, given that these factors significantly influence mental health regardless of the specific school an adolescent attends.

## Data Availability

Data, programming scripts and additional resources linked to this research can be accessed through the zonedo.org portal using the specified digital object identifier (DOI) at: https://doi.org/10.5281/zenodo.15347671.
